# Brazilian oral medicine and oral pathology: international scientific collaborations

**DOI:** 10.4317/medoral.27169

**Published:** 2025-04-06

**Authors:** Luiz Miguel Ferreira, João Pedro Santos Nascimento, Samuel Trezena, Marcos Paulo Maia-Lima, Layanne Ferreira Ribeiro e Sobral, Ana Maria Alencar, Daniella Reis B - Martelli, Márcio Ajudarte Lopes, Fábio Ramoa Pires, Hercílio Martelli-Júnior

**Affiliations:** 1 Department of Oral Diagnosis, School of Dentistry, University of Campinas, FOP-UNICAMP, Piracicaba, São Paulo, Brazil; 2 State University of Montes Claros, UNIMONTES, Montes Claros, Minas Gerais, Brazil; 3 Postgraduate Program in Health Sciences, State University of Montes Claros, UNIMONTES, Montes Claros, Minas Gerais, Brazil; 4 Postgraduate Program in Primary Health Care, State University of Montes Claros, UNIMONTES, Montes Claros, Minas Gerais, Brazil; 5 Oral Pathology and Oral Medicine, School of Dentistry, State University of Montes Claros, UNIMONTES, Montes Claros, Minas Gerais, Brazil; 6 Oral Pathology, Dental School, Rio de Janeiro State University, UERJ; Post-graduation Program in Dentistry, Estácio de Sá University; Rio de Janeiro, Rio de Janeiro, Brazil

## Abstract

**Background:**

Research in Oral Medicine (OM) and Oral Pathology (OP) in Brazil has experienced remarkable growth, gaining international recognition. However, no analysis has evaluated the patterns of evolution of international partnerships and their role in advancing Brazilian research in these areas. This study analyzed collaborations between Brazilian and international researchers in OP/OM.

**Material and Methods:**

A bibliometric cross-sectional survey was conducted using data from Brazilian researchers in OP/OM, identified through the Brazilian Society of Stomatology and Maxillofacial Pathology. Researchers' curriculum on the Lattes platform were analyzed, and data on publications, citations, co-authorships, affiliations, and journals were collected from the Scopus database, focusing on international collaborations. Bibliometric analyses were performed using the Bibliometrix tool in R Studio and VOSviewer software. Statistical trends between decades were compared using the Kruskal-Wallis test.

**Results:**

The sample included 229 researchers, most females (61.6%), affiliated with public institutions (78.9%), and with a mean time since achieving the PhD of 15.27 years. Almost half of the researchers (43.2%) had postdoctoral training, and among them 43.4% completed it abroad, mainly in the United States (USA), Canada, and the United Kingdom (UK). A total of 2,027 articles were analyzed, revealing a 10.53% annual growth in publications. The number of international collaborations significantly increased over decades (*p* < 0.001), with USA, UK, India, and Italy being the leading partners. Collaborative publications showed a significant rise in citations (42.61 per paper), mainly in high-impact journals.

**Conclusions:**

A steady increase in international collaborations in OP/OM was identified, especially with the USA and the UK. These partnerships have increased citations and publications in high-impact journals, which highlights their benefit and importance for research in these areas.

** Key words:**Oral pathology, oral medicine, internationalization, research.

## Introduction

Between July 24 and 27, 2024, it took place in São Paulo, the 50th Brazilian Congress of Stomatology and Oral and Maxillofacial Pathology, commemorating a historic date of the Brazilian Society of Stomatology and Maxillofacial Pathology (SOBEP) (https://www.estomatologia.com.br/congresso-sobep2024). The Congress presented a diverse and very rich program, with approximately 1,050 participants from 20 countries. Among the activities developed in the four days, we highlight the Seminar: "50 years of Stomatology and Oral Pathology in the Brazil: international perspectives", with the following speakers: Dr. Keith Hunter (British Society for Oral and Maxillofacial Pathology - BSOMP), Dr. Mark Lingen (American Academy of Oral and Maxillofacial Pathology - AAOMP), Dr. Nathaniel Treister (American Academy of Oral Medicine - AAOM), Dr. Nikolaos Nikitakis (European Association of Oral Medicine - EAOM), and Dr. Marco Magalhães (University of Toronto) (1). The five speakers highlighted the importance of Brazilian Oral Medicine (OM) and Oral Pathology (OP) in the present and especially in the future in terms of the world scenario.

Brazilian research in OP and OM has witnessed substantial growth and gained international recognition over the past two decades (2,3). This progress is reflected not only in the volume of publications but also in their scholarly impact. Between 2014 and 2023, Brazilian researchers contributed with 4,979 papers, marking a 63% increase in scientific output, which garnered 65,137 citations, a remarkable 65% growth in the same period. These metrics highlight Brazil’s importance in the global research landscape (4).

The active participation of Brazilian researchers in international collaborations has been a key factor in this achievement, facilitating knowledge exchange, resource sharing, and integration into global scientific networks, which enhance research quality and output (5,6). An important example of this in Brazil was the emergence of OP, which began with an international collaboration in 1964, when Professor William G. Shafer conducted an intensive one-month course at the Federal University of Rio de Janeiro, attended by professors from different states, marking a significant milestone in the specialty’s development (7).

In this context, bibliometric analyses are valuable for evaluating research trajectories, productivity trends, collaboration patterns, and thematic focus areas. These metrics are crucial for assessing the scholarly impact of research and its trends, guiding funding decisions, and informing academic promotions (8). However, most studies focus on general indicators like publication and citation counts (2,4,6,9), overlooking deeper analyses of collaboration trends among Brazilian researchers and their international partners. As a result, there is limited understanding of how these collaborations have evolved or contributed to the growth of OP and OM, particularly in quality and visibility.

Therefore, this study aimed to analyze collaborations between Brazilian and international researchers in OP and OM, focusing on identifying the countries with the highest frequency of partnerships with Brazil and the changes that have occurred in these collaborations over the years.

## Material and Methods

- Design and Participants

This cross-sectional study was based on publicly available secondary data, which did not require approval from a Research Ethics Committee. Initially, a database was compiled of 533 professionals who were active members of SOBEP in 2024 (www.estomatologia.com.br). The curriculum vitae (CVs) of each selected researcher were accessed through the Lattes platform (https://lattes.cnpq.br/) in October 2024 and analyzed to apply the exclusion criteria. From the initial group, 304 individuals were excluded for the following reasons: 203 had not completed postgraduate studies in OP and/or OM, 41 had not updated their CVs in the last 1.5 years; 4 were retired; 6 were not researchers in OP and/or OM; 5 were not Brazilian or did not work professionally in Brazil; and 45 did not have a consistent publication history. As a result, the final sample consisted of 229 professionals.

- Data collection

Based on the information available in the CVs, the following data were extracted to create a profile of the group of researchers: gender, institution of origin, administrative category of the institution, geographical distribution, institution where the PhD was completed, time since PhD conclusion, completion of a postdoctoral fellowship, and the country where the postdoctoral fellowship was conducted.

The researchers' profiles were accessed in November 2024 via the Scopus database (https://www.scopus.com/), selected for its large number of indexed journals (10). All scientific articles indicating international collaborations were selected. The data from the documents were exported and contained complete information on the authors, affiliations, year of publication, journal of origin, title, subject categories, references, abstract and citations. However, the variables of interest for this study were only the number of scientific articles published per year, the number of citations received per article, the involvement of co-authors from other countries, affiliations, and the name of the journals where the articles were published. This data was used for the bibliometric analysis. All records identified as scientific materials were included in the analysis.

- Bibliometric analysis

Subsequently, the list of included papers was analyzed in R Studio (version 2024.09.1) to remove duplicates. The Bibliometrix tool, available in this software, was used to visualize the number of scientific articles, citations and collaborations per year. The main journals were also obtained. The bibliometric analysis software VOS viewer (version 1.6.20) was applied to visualize the clusters between the countries that collaborate in Brazilian studies. In addition, to enrich the analysis, the impact factors and access status of the journals were collected using the Journal Citation Reports (JCR) (https://clarivate.com/academia-government/scientific-and-academic-research/research-funding-analytics/journal-citation-reports/).

- Statistical analysis

The database was created and statistical analysis was conducted using SPSS (Statistical Package for Social Sciences for Windows®, Inc., USA) version 27.0. Categorical variables were analyzed through absolute and relative frequencies, while numerical variables were described using the mean and standard deviation. To check for normality, the Kolmogorov-Smirnov test was applied to these variables. To compare the number of papers, citations, and international collaborations over time, data were grouped into 10-year intervals and analyzed using the Kruskal-Wallis test. A *p-value* of < 0.05 was considered statistically significant.

## Results

Of the 229 participants in this study, the majority were female (*n*=141/61.6%) and worked in public institutions (*n*=180/78.9%). The mean time since receiving the PhD was 15.27 years (95% CI 14.09-16.44). Of the researchers, 99 (43.2%) completed postdoctoral training, and of these, 43.4% (*n*=43) conducted in other countries. The countries were: the United States (*n*=23), Canada (*n*=6), United Kingdom (*n*=5), Germany (*n*=2), Spain (*n*=2), Sweden (*n*=1), Finland (*n*=1), Mexico (*n*=1), Australia (*n*=1), and Belgium (*n*=1).

Slightly more than half of the researchers were concentrated in the Southeast region of the country (*n*=121, 52.8%). Of these, 75 (32.8%) resided in the state of São Paulo, followed by the state of Minas Gerais (*n*=25, 10.9%). The Northeast region had 55 researchers (24%), followed by the South region (*n*=34/14.8%) and the Midwest (*n*=15/6.6%). Only 4 (1.7%) researchers were from the North region of the country.

By accessing the profiles of all the researchers, 14,524 articles were identified in the Scopus database. By applying the filter by country, the number of papers was reduced to 3,360, which were exported. After removing duplicates, 2,027 scientific articles remained. The publication period ranged from 1971 to November 2024. Table 1 presents the number of papers published by researchers and the corresponding number of citations, distributed by decades. The annual growth rate of publications was 10.53% and the average citations per article was 42.61. A total of 86,364 citations were recorded (SD ± 1,599.33).

The top 10 journals identified in the sample accounted for 583 articles (28.76% of total publications). Including 135 in the Journal of Oral Pathology and Medicine (IF 2.7); 106 in Oral Diseases (IF 2.9); 106 in Oral Surgery, Oral Medicine, Oral Pathology and Oral Radiology (IF 2.0); 54 in Oral Oncology (IF 4.0); 39 in Head and Neck Pathology (IF 3.2); 39 in Medicina Oral, Patología Oral y Cirugía Bucal (IF 1.8); 28 in Clinical Oral Investigations (IF 3.1); 27 in Supportive Care in Cancer (IF 2.8); 25 in PLoS One (IF 2.9); and 24 in Brazilian Oral Research (IF 1.5). Although not included in the top 10, some manuscripts were published in journals with high impact factors, including 16 in The Lancet (IF 98.4); 21 in the Journal of Periodontology (IF 4.2); and 16 in Scientific Reports (IF 3.8).

**Figure 1:**
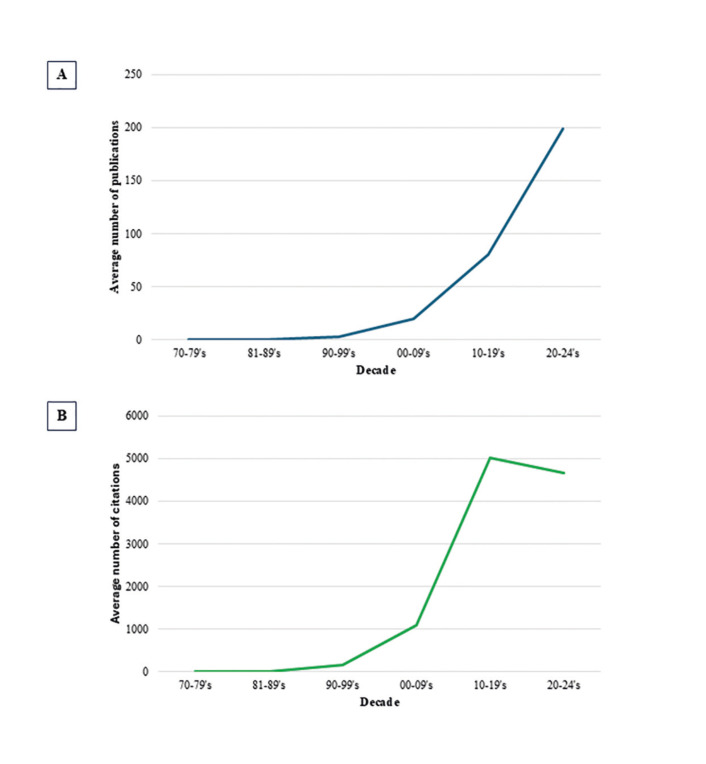
Trends in collaborations in publications and citations in Oral Pathology and Oral Medicine by decade. (A) Average number of scientific publications per decade. (B) Average number of citations per decade.

Between 1971 and 1979, the average number of publications was only 0.11, while in the following decades, especially after 2000, an exponential growth was observed. The increase in citations followed a similar trend, with an average of 0.33 citations per publication in the 1970s, rising to 4,671.20 in the 2020-2024 decade. Statistically significant differences (*p* < 0.001) were observed between the decades, highlighting the growing impact of scientific publications over time, as shown in Fig. 1.

Table 2 shows the average annual collaborations per publication. A statistically significant association (*p* < 0.001) was identified in all analyzed countries, indicating that the number of international collaborations consistently increased over the decades. The United States and Denmark were the countries that initially began collaborating with Brazilians. In the most recent decade analyzed, the countries with the highest average collaborations were the United States, the United Kingdom, India, Italy, and Australia. The largest number of foreign participations were identified from the 2010-2019’s decade.

The University of Washington and the University of Michigan were the institutions that collaborated the most with Brazilian researchers, with participation in 841 and 515 papers, respectively. Following them, Imperial College London, University College London, and the University of Sheffield stood out as the leading institutions from the United Kingdom, with participation in 185, 149, and 129 scientific articles, respectively.

**Figure 2:**
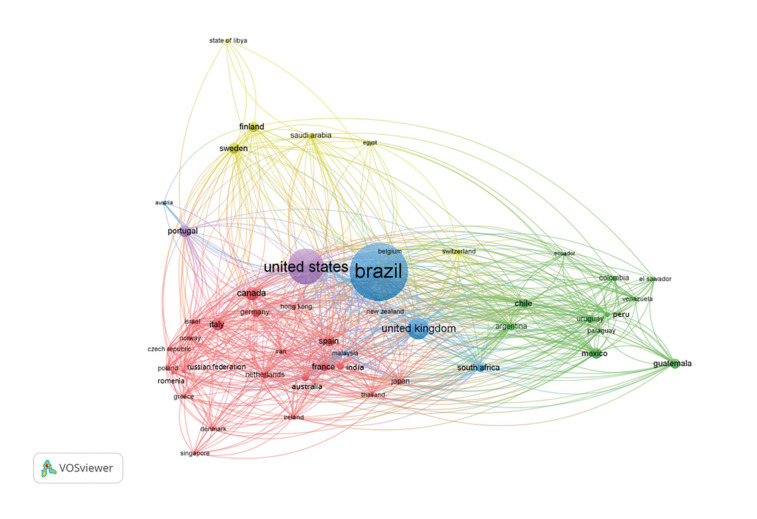
Network of international collaborations in Oral Pathology and Oral Medicine. The size of the circles reflects the number of publications associated with each country, while the lines represent the strength of the collaborative connections between them.

Fig. 2 illustrates the map of international collaborations and Brazil's connection with major research Centers in OP and OM. It is evident that the United States and the United Kingdom are the main collaboration partners of Brazilian researchers, as indicated by the larger size of the circles associated with these countries.

Significant collaborations are also noted with Portugal, Canada, South Africa, Guatemala, Mexico, Chile, Italy, France, and Spain. Additionally, less frequent interactions with countries such as Egypt, El Salvador, and Libya are also observed. The Figure further suggests the formation of two well-defined collaboration networks, one predominantly composed of European countries and the other of Latin American countries. The broad global connectivity observed reflects the growing internationalization of publications in these fields, highlighting the existence of multiple interconnected networks.

Discussion 

This is the first study to analyze international collaborations in Brazilian research in OP and OM. We present updated information on the countries, papers, citations, institutions, and journals with the greatest impact. The analysis confirmed the group’s high scientific productivity, with significant participation in international collaborations. Notably, there is a considerable number of scientific articles published in journals indexed in relevant databases, such as Scopus.

Our analysis revealed a predominance of female researchers (1.6:1), contrasting with our previous study, which identified a predominance of male researchers (1.27:1) (4). This result appears to contradict the trend highlighted by Handelsman *et al*. (2005) (11), who emphasized the persistence of gender inequality and the historical underrepresentation of women in science. The discrepancy between these findings may be related to the increase in membership of SOBEP compared to the previous year (12).

The geographical distribution of the researchers analyzed was heterogeneous, with 52.8% working in the Southeast region of the country, while only 1.7% were based in the North region. This pattern has also been identified in other studies, which highlight the predominance of researchers, particularly in the states of São Paulo and Minas Gerais, as also observed in the present study (4,13). Another factor to highlight is that most of the master's and PhD programs are concentrated in the Southeast region, where the postgraduate programs with the highest ratings are also located (https://sucupira.capes.gov.br/sucupira/). This may explain the predominance of researchers in this region. Most of the institutions were public (78.9%), which is consistent with the high number of publications by these researchers, considering that a large portion of Brazilian researchers are affiliated with graduate programs at public universities, responsible for about 85% of the country's total scientific production (www.gov.br/capes/pt-br).

The average time of researchers' careers since obtaining their doctoral degree was 15.27 years. In this regard, it is worth highlighting that longer careers enable researchers to build broader and more robust networks, which contributes to an increase in the number of publications (14,15). Among the researchers analyzed, 43 (43.4%) completed postdoctoral training in other countries. There was a noticeable compatibility between the countries where the training took place and those that had many collaborations with Brazil, such as the United States (n = 23), Canada (*n*=6), and the United Kingdom (*n*=5). This international training enables participation in multicenter international projects, promoting the acquisition of new scientific knowledge, the creation of research groups, and the exchange of methodological experiences (16).

The results of this study highlight a significant increase (*p* < 0.001) in scientific production with international partnerships in OP and OM over the decades, as evidenced by the growth in both the number of publications and the impact of these studies. Since the 2000s, this growth has intensified, reaching an average of 198.80 publications and 4,671.20 citations in the 2020-2024 decade. This trend aligns with the findings of a recent study, which observed a 63% increase in the number of citations received by 50 Brazilian researchers between 2014-2023 compared to the 2004-2013 period (4). This progress underscores the crucial role of international collaborations, which enhance the visibility of research and can triple the number of citations, driven by greater relevance and the geographic diversification provided by these partnerships (17). These consequences stem from the ability to combine resources, expertise, and technological capabilities, and provide access to diverse population data, which raises the value of the evidence generated (18).

The analysis of publications by journal revealed that the top 10 journals accounted for 28.76% of the articles, with a particular emphasis on ‘Journal of Oral Pathology and Medicine, Oral Diseases and Oral Surgery, Oral Medicine, Oral Pathology and Oral Radiology’. This result supports the study by De Andrade *et al*. (2018) (2), which identified that a significant portion of Brazilian scientific production was published in six international journals, the same ones found to be the most frequent in this study. These data reflect the quality of international multicentric collaborations. In this context, Farias *et al*. (2024) (6) highlighted in their recent study that 36 researchers in OP and OM, National Council for Scientific and Technological Development (CNPq) productivity fellows, have published 6,313 articles in international journals throughout their careers, demonstrating a significant increase in indicators of internationalization of Brazilian researchers' scientific output.

The analysis of collaborations by country showed a significant increase for all countries included through the decades. The USA, UK, India and Italy were the countries with the greatest average collaborations in the last decade analyzed. All the four countries are in the top 10 of global production ranking (https://ncses.nsf.gov/pubs/nsb202333/Table/PBS-1) and in the top 5 of global international collaborations ranking (https://ncses.nsf.gov/pubs/nsb202333/international-collaboration-and-citations).

The internationalization of Brazilian research is supported by national policies and programs, such as the Institutional Internationalization Program (PrInt) of the Coordination for the Improvement of Higher Education Personnel (CAPES) and the Graduate Student Agreement Program (PEC-PG), which promote global partnerships, scholarships and academic exchanges, especially with countries in the Global South (https://www.gov.br/capes/pt-br). In addition, research funding agencies such as CNPq, CAPES and other regional agencies play crucial roles in this context (19), offering comprehensive support covering master's, doctoral and post-doctoral levels, including specific initiatives aimed at mobility and international collaborations (https://www.gov.br/capes/pt-br/acesso-a-informacao/acoes-e-programas/bolsas/bolsas-e-auxilios-internacionais). NoTable examples include the Science without Borders program, launched in 2011, which, with an investment of over US$ 2 billion, has enabled around 101,000 Brazilian students and researchers to develop international academic experiences, strengthening global collaboration (20). Despite these significant efforts, Brazil still faces challenges such as financial constraints, inconsistent funding and limited infrastructure, which hinder the country's full integration into the global scientific community (21,22). Overcoming these barriers is indispensable for realizing the benefits of internationalization and consolidating Brazil's position in global research.

In Brazil, emerging technologies such as telepathology and telemedicine have begun to reshape the landscape of education and research in OP and OM. Due to the extensive territorial dimension and unequal distribution of human resources in OP/OM, telemedicine strengthens interactions between remote regions and major academic centers (23-25). The exchange of knowledge and matrix support is important not only in diagnostic support, but in the creation of scientific partnerships between institutions that present foreign partnerships, favoring multicenter collaborations at a national and international level.

This study has some limitations. One of them is that only SOBEP members were included in the analysis. Although the vast majority of qualified researchers in Brazil in the areas of OP and OM are members of SOBEP, it is possible that some are not. Another limitation refers to the use of public data from the Lattes platform and the Scopus database, which may contain incomplete or outdated information in researchers' profiles or publication records. Furthermore, the use of bibliometric tools, although useful for analyzing trends and collaborations, does not consider the qualitative aspects of scientific contributions. These limitations, however, are not exclusive to this study, but are inherent to the methodology used in most bibliometric analyses. It is therefore essential that researchers fill in the data correctly when submitting articles and keep their CVs up to date to ensure greater accuracy in future analyses.

We would like to take this opportunity to pay tribute to some researchers who have collaborated significantly with Brazilian researchers in OP and OM even before these specialties were recognized in the country. OP was recognized as a specialty in 1971 and OM in 1992 (https://website.cfo.org.br/). However, long before these dates, Professor William G. Shafer was already active in these fields, making remarkable contributions to the advancement of oral diagnosis in Brazil. Another noTable example is Professor Dr. Sol Silverman Jr. from the University of California, San Francisco. He participated as a speaker in a training course held in 1969, sponsored by the W. K. Kellogg Foundation. This event took place at the University of São Paulo and the AC Camargo Cancer Center. During the course, Dr. Silverman Jr. shared his experience in OM with professors from various Brazilian institutions, promoting the development of the field in the country (3).

In conclusion, this study highlights the substantial progress made by Brazilian researchers in OP and OM on the international stage. Over the last two decades, international collaborations have increased significantly, particularly with important countries on the global research scene, such as the USA and the UK. These partnerships have contributed to remarkable growth in scientific productivity and citation metrics, particularly in high-impact journals.

## Figures and Tables

**Table 1:** Comparison of average yearly publications and citations across decades.

Comparison	Decades	Number	Mean	SD	*p*-value
Publications	71-79's	1	0.11	0.33	<0.001*
	80-89's	1	0.10	0.31	
	90-99's	29	2.90	2.60	
	00-09's	195	19.50	9.32	
	10-19's	807	80.70	33.21	
	20-24's	994	198.80	15.12	
Citations	70-79's	3	0.33	1.00	<0.001*
	80-89's	102	10.20	32.25	
	90-99's	1,666	166.60	147.99	
	00-09's	11,025	1,102.50	571.56	
	10-19's	50,212	5,021.20	3,614.37	
	20-24's	23,356	4,671.20	6,078.97	

SD: Standard Deviation. *Statistical association (*p*<0.05) using Kruskal-Wallis test.

**Table 2:** Average annual collaborations per publication by country and decade.

Country		Decades						p-value*
		71-79's Mean (SD)	80-89's Mean (SD)	90-99's Mean (SD)	00-09's Mean (SD)	10-19's Mean (SD)	20-24's Mean (SD)	
Africa	Egypt	0	0	0	0	0.2 (0.31)	0.98 (0.06)	<0.001*
	Nigeria	0	0	0	0.01 (0.01)	0.17 (0.27)	0.91 (0.05)	<0.001*
	South Africa	0	0	0	0.02 (0.04)	0.46 (0.55)	1.84 (0.27)	<0.001*
America	Argentina	0	0	0	0.01 (0.01)	0.24 (0.26)	0.78 (0.19)	<0.001*
	Canada	0	0	0	0.32 (0.22)	1.46 (0.64)	2.97 (0.31)	<0.001*
	Chile	0	0	0	0.01 (0.01)	0.26 (0.26)	0.82 (0.21)	<0.001*
	Colombia	0	0	0	0.01 (0.01)	0.22 (0.21)	0.79 (0.08)	<0.001*
	Guatemala	0	0	0	0.14 (0.2)	0.66 (0.09)	0.55 (0.07)	<0.001*
	Mexico	0	0	0	0.19 (0.19)	0.82 (0.38)	1.78 (0.22)	<0.001*
	Peru	0	0	0	0.01 (0.02)	0.25 (0.14)	0.58 (0.08)	<0.001*
	United States	0.11 (0.33)	0.3 (0.94)	6.53 (5.5)	11.03 (2.74)	12.44 (3.52)	21.86 (1.58)	<0.001*
	Uruguay	0	0	0	0	0.05 (0.08)	0.31 (0.12)	<0.001*
Asia	Bangladesh	0	0	0	0	0.08 (0.15)	0.45 (0.03)	<0.001*
	China	0	0	0	0.01 (0.01)	0.95 (1.25)	3.28 (0.37)	<0.001*
	India	0	0	0	0.01 (0.01)	1.34 (2.04)	5.88 (0.46)	<0.001*
	Israel	0	0	0	0.09 (0.02)	0.28 (0.19)	0.57 (0.07)	<0.001*
	Japan	0	0	0.21 (0.3)	0.15 (0.04)	0.61 (0.5)	1.61 (0.18)	<0.001*
	Korea	0	0	0	0	0.24 (0.32)	0.86 (0.07)	<0.001*
	Malaysia	0	0	0	0.02 (0.04)	0.35 (0.4)	1.07 (0.14)	<0.001*
	Nepal	0	0	0	0	0.1 (0.19)	0.55 (0.49)	<0.001*
	Pakistan	0	0	0	0	0.17 (0.3)	0.89 (0.05)	<0.001*
	Saudi Arabia	0	0	0	0	0.18 (0.32)	1.06 (0.07)	<0.001*
	Singapore	0	0	0	0	0.37 (0.46)	0.97 (0.1)	<0.001*
	Turkey	0	0	0	0.09 (0.06)	0.31 (0.21)	1.23 (0.59)	<0.001*
Europe	Austria	0	0	0	0	0.2 (0.27)	0.64 (0.11)	<0.001*
	Croatia	0	0	0	0	0.14 (0.22)	0.57 (0.09)	<0.001*
	Czech Republic	0	0	0	0.01 (0.05)	0.29 (0.07)	0.53 (0.15)	<0.001*
	Belgium	0	0	0	0	0.4 (0.46)	1.2 (0.22)	<0.001*
	Denmark	0	0.1 (0.31)	0.36 (0.36)	0.06 (0.03)	0.46 (0.59)	1.62 (0.31)	<0.001*
	Finland	0	0	0	0.08 (0.08)	1.67 (1.12)	3.18 (0.32)	<0.001*
	France	0	0	0	0.02 (0.03)	1.11 (0.89)	2.38 (0.32)	<0.001*
	Germany	0	0	0.64 (0.91)	0.38 (0.14)	1.2 (0.97)	3.22 (0.38)	<0.001*
	Greece	0	0	0	0	0.23 (0.29)	0.93 (0.19)	<0.001*
	Ireland	0	0	0	0	0.21 (0.24)	0.69 (0.18)	<0.001*
	Italy	0	0	0	0.24 (0.26)	1.62 (1.34)	5.5 (1.39)	<0.001*
	Lithuania	0	0	0	0	0.11 (0.16)	0.44 (0.1)	<0.001*
	Netherlands	0	0	0	0.33 (0.22)	0.77 (0.74)	2.22 (0.4)	<0.001*
	Norway	0	0	0.09 (0.19)	0.19 (0.09)	0.39 (0.46)	1.31 (0.17)	<0.001*
	Poland	0	0	0	0	0.64 (0.81)	1.91 (0.25)	<0.001*
	Portugal	0	0	0	0.1 (0.17)	1.25 (0.49)	2.91 (0.76)	<0.001*
	Romania	0	0	0	0	0.17 (0.29)	0.95 (0.1)	<0.001*
	Serbia	0	0	0	0	0.09 (0.13)	0.43 (0.03)	<0.001*
	Spain	0	0	1.31 (2.22)	1.33 (0.6)	1.53 (1.02)	3.87 (0.95)	<0.001*
	Sweden	0	0	0	0.19 (0.09)	0.83 (0.71)	2.19 (0.23)	<0.001*
	Switzerland	0	0	0	0.05 (0.05)	0.47 (0.39)	1.12 (0.18)	<0.001*
	United Kingdom	0	0	2.27 (2.25)	4.31 (0.89)	4.96 (1.48)	14.71 (6.63)	<0.001*
Oceania	Australia	0	0	0	0.06 (0.03)	0.91 (1.3)	4.48 (0.39)	<0.001*
	New Zealand	0	0	0	0	0.27 (0.3)	0.88 (0.23)	<0.001*

*Statistical association (p<0.05) using Kruskal-Wallis test
